# The importance of clinic attendance in the first six months on antiretroviral treatment: a retrospective analysis at a large public sector HIV clinic in South Africa

**DOI:** 10.1186/1758-2652-13-49

**Published:** 2010-12-07

**Authors:** Alana T Brennan, Mhairi Maskew, Ian Sanne, Matthew P Fox

**Affiliations:** 1 Center for Global Health and Development, Boston University, Boston, MA, USA; 2 Health Economics and Epidemiology Research Office, Johannesburg, South Africa; 3 University of Witwatersrand, Johannesburg, South Africa; 4 Right to Care, Johannesburg, South Africa; 5 Clinical HIV Research Unit, Department of Medicine, Faculty of Health Sciences, University of the Witwatersrand, South Africa; 6 Department of Epidemiology, Boston University School of Public Health, Boston, MA, USA

## Abstract

**Background:**

Adherence to care and treatment are essential for HIV-infected individuals to benefit from antiretroviral therapy (ART). We sought to quantify the effects on treatment outcomes of missing visits soon after initiating ART.

**Methods:**

We analyzed data from HIV-infected patients initiating ART at Themba Lethu Clinic, Johannesburg, South Africa, from April 2004 to August 2008. We used log-binomial regression to evaluate the relative risk of missing visits during the first six months of ART on immunological response and virologic suppression. Cox models were used to evaluate the relationship between missed visits and mortality and loss to follow up over 12 months.

**Results:**

Of 4476 patients, 65% missed no visits, while 26% missed one visit, 7% missed two and 1.6% missed three or more visits during the first six months on treatment. Patients who missed three or more medical or antiretroviral (ARV) visits had a two-fold increased risk of poor CD4 response by six months, while the risk of failing to achieve virologic suppression by six months increased two- to five-fold among patients who missed two and three or more medical or ARV visits. Adjusted Cox models showed that patients who missed two (HR 2.1; 95% CI: 1.0-4.3) and three or more (HR 4.7; 95% CI: 1.4-16.2) medical visits had an increased risk of death, while those who missed two ARV (HR 3.8; 95% CI: 2.5-5.8) or three or more medical (HR 3.0; 95% CI: 1.1-8.1) visits had an increased risk of loss to follow up.

**Conclusions:**

Thirty-five percent of patients missed one or more visits in the first six months on treatment, increasing their risk of poorer outcomes. These patients could be targeted for additional adherence counselling to help improve ART outcomes.

## Background

Expansion of antiretroviral therapy (ART) treatment programmes in resource-limited settings has helped to substantially improve patient outcomes on ART [1]; however, programmatic outcomes, such as death and loss to follow up, still remain high when compared with industrialized countries [2-4].

In order to help improve overall treatment outcomes, treatment providers need to focus retention efforts on ART patients who are at increased risk of poor clinical outcomes and becoming lost to follow up. HIV clinics in resource-limited settings continue to struggle to keep patients in care and adhering to treatment in the early stages of ART [4-8], with high mortality among patients who leave care [9,10]. Adhering to the required treatment schedule early on in care can be difficult, but may be an important step in maintaining long-term retention, adherence and reductions in morbidity and mortality. Previous studies have shown that poor adherence to treatment regimens [11-13] and medical appointments soon after initiating treatment can decrease the overall effectiveness of ART [14-21], but it is not clear if these early missed visits have any longer term effects.

Because late presentation for ART is so common [22], identifying patients who are not attending scheduled clinic appointments on time and developing strategies directed at keeping them in care and adhering to treatment is critical to improving long-term outcomes. Although several studies have looked at the association between missed visits and patient outcomes, few have evaluated this relationship in a resource-limited setting and none have looked at the long-term effects of missed visits early on in treatment. We hypothesized that those HIV-positive patients, who miss visits in the first six months of treatment, but return to care, will be at increased risk of death and loss to follow up, and have poorer immunological and virologic outcomes when compared with patients with perfect appointment attendance.

## Methods

### Cohort description

This retrospective cohort study was conducted in the Themba Lethu Clinic in Johannesburg, South Africa. Themba Lethu is one of the largest ART clinics in South Africa, with more than 26,500 patients enrolled in care since April 2004, more than 17,700 of whom have initiated ART. The clinic staff provides care according to South African national Department of Health guidelines [23]. Patient data used in this analysis is extracted from an electronic patient record system, called TherapyEdge-HIV™.

Use of Themba Lethu Clinic data was approved by the Human Research Ethics Committee of the University of the Witwatersrand. Approval for analysis of the data in a de-identified manner was granted by the Institutional Review Board of Boston University.

### Eligibility criteria

Our analysis included HIV-positive patients who were eligible for ART based on the 2004 South African national treatment guidelines [23]. Eligible subjects were ART naïve and ≥18 years of age, with a baseline CD4 count at ART initiation of ≤200 cells/mm^3 ^and initiated onto standard government first-line ART regimens of stavudine (d4T) or zidovudine (AZT) with lamivudine (3TC) and either efavirenz (EFV) or nevirapine (NVP) between April 2004 and August 2008. We further excluded pregnant women, those who had less than three scheduled medical and three scheduled antiretroviral (ARV) pickup visits during the first six months of ART, and patients with less than 21 months of potential follow-up time (see section on person-time).

### Study variables

We evaluated the relationship between missed medical and ARV pickup visits within the first six months after initiating ART on four outcomes: (1) mortality; (2) loss to follow up; (3) immunologic response; and (4) virologic suppression. Loss to follow up was defined as not having been to the clinic for at least four months. Data on mortality, including for those patients who are lost to follow up, is verified with the South African National Vital Registration Infrastructure Initiative [24-26]. Poor immunological response was defined as an increase of <50 cells/mm^3 ^by six months on ART, while failure to achieve virologic suppression was defined as a viral load of ≥400 copies/ml by six months on ART. When CD4 values were missing, they were interpolated by taking the mean of the proceeding and following measures when both were available (6%).

Missing medical and ARV pickup visits during the first six months of initiating first-line ART was the primary independent variable. After ART initiation, ARV visits are scheduled monthly, while medical visits are booked at months one, four and five. Patients prescribed nevirapine are scheduled for two additional medical visits at two weeks and two months to monitor for liver toxicity. Thus, in the first six months on treatment, patients can have up to five ARV visits (the first is excluded as it is the date of ART initiation) and between three and five scheduled medical visits. Unscheduled visits were not counted in the exposure variable since the exposure of interest is missing a scheduled visit, so any non-scheduled visit could not be missed.

We determined each participant's missed visit status by evaluating appointment attendance records. Appointment scheduling is managed with the TherapyEdge-HIV™ database, which is used to schedule all visits and to record the actual date on which a scheduled visit was completed. As patients are given two extra days worth of pills each month in case they cannot attend their scheduled ARV visits on time, it is thus likely that in the first six months on treatment, patients who are more than seven days late for a pickup would be without medication. Accordingly, we defined a missed visit as being at least seven days late for that scheduled visit. For our primary analysis, missed visit status over the first six months of initiating ART was categorized as patients who missed 0, 1, 2 and ≥3 visits. We analyzed missed visits stratified by type of visit as either medical or ARV drug pickup.

For death and loss to follow up analyses (the only two time-to-event analyses), person-time accrued from nine months after treatment initiation. We chose the period of nine months in order to exclude any deaths or losses to follow up that occurred during exposure (missed visits in the first six months on ART). We excluded an additional three months in order to reduce the impact of reverse causality (i.e., death causing patients to miss visits). Person-time accrued until the earliest of: (1) date of death; (2) date of loss to follow up; (3) date of transfer; or (4) completion of 12 additional months of follow up.

### Statistical analysis

Descriptive statistics were performed to look for baseline differences in the distribution of missed visits. Log-binomial regression was used to evaluate predictors associated with missing a visit during the first six months of initiating ART. We also estimated the relative risk of missing a visit in the first six months on both poor CD4 response (increase of <50 cells/mm^3^) and failure to achieve virologic suppression (< 400 copies/mL) by six months on ART. Log-binomial models were adjusted for age, sex, WHO stage III/IV condition, body mass index (BMI), baseline haemoglobin and baseline CD4 count (categorized ≤50, 51-100 and 101-200 cell/mm^3^) and baseline ART regimen (AZT vs. d4T and NVP vs. EFV). We estimated crude and adjusted hazard ratios of mortality and loss to follow up by missed visit status using Cox proportional hazards models. Hazard models were also adjusted for age, sex, baseline regimen, total number of scheduled visits and CD4 count by the ninth month of ART.

## Results

Out of 10,048 patients aged 18 years or older who initiated ART between April 2004 and August 2008 with CD4 counts of <200 cells/mm^3^, we excluded 1292 not on standard first-line ART, 1205 non-naïve patients, 109 women pregnant at baseline, 1875 patients who did not have the necessary six months of exposure or died prior to nine months of ART, and 1091 patients who did not have at least three medical and three scheduled ARV visits in the first six months of treatment. This left 4476 patients eligible for this analysis.

Baseline demographics and clinical characteristics show that patients had a median baseline CD4 count of 76 cells/mm^3 ^(IQR 28-136 cells/mm^3^), were predominately female (62.8%), had a median age of 36.1 years (IQR 31.3-42.2), and were typically prescribed d4T-3TC-EFV (89.4%) as a baseline regimen (Table 1). Those who missed 1, 2 or ≥3 visits (ARV or medical) in the first six months on ART were similar in baseline demographic and clinical characteristics to those who missed none, with the exception of having a higher proportion of men. Additionally, patients who missed ≥3 visits had a higher percentage of patients older than 25 years and more patients on NVP- and AZT- based regimens when compared with the other three groups.

**Table 1:** Baseline characteristics and outcomes of patients attending an HIV clinic in Johannesburg, South Africa, stratified by missed visit status in the first six months of ART (n = 4476)

	Missed visit status (Medical or ARV)				
**Characteristics**	**0**	**1**	**2**	**≥ 3**	**Total**
	**(n = 2913)**	**(n = 1178)**	**(n = 312)**	**(n = 73)**	**(n = 4476)**
	**N (%)**	**N (%)**	**N (%)**	**N (%)**	**N (%)**

Sex					
Female	1870 (64.2%)	724 (61.5%)	179 (57.4%)	37 (50.7%)	2810 (62.8%)
Male	1043 (35.8%)	454 (38.5%)	133 (42.6%)	36 (49.3%)	1666 (37.2%)
					
Age at ART initiation					
18-24.9	125 (4.3%)	60 (5.1%)	13 (4.2%)	7 (9.6%)	205 (4.6%)
25-29.9	427 (14.7%)	165 (14.0%)	50 (16.0%)	12 (16.4%)	654 (14.6%)
30-39.9	1390 (47.7%)	561 (47.6%)	149 (47.8%)	35 (48.0%)	2135 (47.7%)
40-49.9	714 (24.5%)	287 (24.4%)	79 (25.3%)	14 (19.2%)	1094 (24.4%)
≥ 50	257 (8.8%)	105 (8.9%)	21 (6.7%)	5 (6.9%)	388 (8.7%)
					
CD4 at ART initiation (cells/mm^3^)					
0-50	1069 (36.7%)	433 (36.8%)	121 (38.8%)	26 (35.6%)	1649 (36.8%)
51-100	680 (23.3%)	266 (22.6%)	62 (19.9%)	16 (21.9%)	1024 (22.9%)
101-200	1123 (38.6%)	464 (39.4%)	128 (41.0%)	30 (41.1%)	1745 (39.0%)
Missing	41 (1.4%)	15 (1.3%)	1 (0.3%)	1 (1.4%)	58 (1.3%)
					
WHO stage at ART initiation					
I/II	1485 (51.0%)	570 (48.4%)	169 (54.2%)	33 (45.2%)	2257 (50.4%)
III	1183 (40.6%)	496 (42.1%)	122 (39.1%)	33 (45.2%)	1834 (41.0%)
IV	245 (8.4%)	112 (9.5%)	21 (6.7%)	7 (9.6%)	385 (8.6%)
					
First-line ART regimen					
d4T/3TC/EFV	2614 (89.7%)	1053 (89.4%)	280 (89.7%)	55 (75.3%)	4002 (89.4%)
d4T/3TC/NVP	219 (7.5%)	95 (8.1%)	24 (7.7%)	13 (17.8%)	351 (7.8%)
AZT/3TC/EFV	75 (2.6%)	28 (2.4%)	8 (2.6%)	5 (6.9%)	116 (2.6%)
AZT/3TC/NVP	5 (0.2%)	2 (0.2%)	0 (0%)	0 (0%)	7 (0.7%)
					
Outcomes by sixth month of ART					
Poor CD4 response (increase <50 cells/mm^3^)	509 (17.5%)	230 (19.5%)	60 (19.2%)	21 (28.8%)	820 (18.3%)
Failure to achieve VL suppression (< 400copies/mL)	191 (6.6%)	80 (6.8%)	34 (10.9%)	11 (15.1%)	316 (7.1%)
					
Outcomes by 12 months of follow up (21 months of ART)					
Death	71 (2.4%)	32 (2.7%)	6 (2.0%)	6 (8.2%)	115 (2.6%)
Loss to follow up	174 (6.0%)	65 (5.5%)	27 (8.7%)	11 (15.1%)	277 (6.2%)
Transferred	60 (2.1%)	37 (3.1%)	6 (2.0%)	1 (1.0%)	104 (2.3%)
Alive and in care	2608 (89.5%)	1044 (88.6%)	273 (87.5%)	55 (75.3%)	3980 (89.0%)

**Characteristics**	**0**	**1**	**2**	**≥ 3**	**Total**
	Median (IQR)	Median (IQR)	Median (IQR)	Median (IQR)	Median (IQR)

CD4 at ART initiation (cells/mm^3^)	76 (29-137)	77 (28-136)	72 (25-136)	82.5 (20.5-133.5)	76 (28-136)
					
CD4 nine months after ART initiation (cells/mm^3^)	245 (175-334	238 (176-323)	240 (177-303)	221 (151-319)	243 (175-328)
					
Time on ART (months)	28.4 (11.8-33.3)	30.3 (18.0-36.3)	30.8 (19.8-39.5)	25.6 (15.4-40.2)	25.6 (15.4-50.2)
					
Hb at ART initiation (ug/dL)	11.4 (10.0-12.9)	11.5 (10.1-13.0)	11.7 (9.9-13.1)	11.7 (10.3-13.0)	11.5 (10.0-12.9)
					
BMI at ART initiation	21.4 (19.0-24.4)	21.2 (18.8-24.3)	21.4 (19.4-24.2)	20.6 (18.6-22.6)	21.4 (19.0-24.3)
					
Age at ART initiation	36.1 (31.3-42.2)	36.1 (31.4-42.1)	35.4 (31.5-42.1)	35.7 (29.5-40.0)	36.1 (31.3-42.2)
					
Total medical visits scheduled in first six months of ART	4 (4-5)	4 (3-5)	4 (3-5)	4 (3-5)	4 (3-5)
					
Total ARV visits scheduled in first six months of ART	5 (4-6)	5 (4-5)	5 (4-5)	4 (4-5)	5 (4-5)

ART, antiretroviral therapy; ARV, antiretroviral; Hb, haemoglobin; BMI, body mass index; d4T, stavudine; 3TC, lamivudine; EFV, efavirenz; NVP, nevirapine; IQR, interquartile range

Among 4476 patients initiating ART, 65.1% (n = 2913) attended all scheduled medical and ARV visits, 1178 (26.3%) missed one visit, 312 (7.0%) missed two visits, and 73 (1.6%) missed three or more visits during the first six months on treatment. Patients had a median of four (IQR 3-5) scheduled medical visits and a median of five (IQR 4-5) scheduled ARV pickup visits. There were a total of 115 deaths (2.6%) and 277 patients were lost to follow up (6.2%) during the 12 months of observation. Median follow-up time (which began nine months after ART initiation) among patients who died and missed 0, 1, 2 and ≥3 visits (medical or ARV) was 4.5 months (IQR 1.8-9.2), 5.7 months (IQR 2.8-9.8), 3.5 months (IQR 2.0-6.8) and 2.2 months (IQR 0.6-3.5), respectively. Median follow-up time among patients who were lost to follow up and who missed 0, 1, 2 and ≥3 visits (medical or ARV) was 6.5 months (IQR 3.8-8.8), 7.1 months (IQR 3.9-9.4), 5.4 months (IQR 3.2-7.6) and 6.3 months (IQR 3.2-8.9), respectively.

### Predictors of missed visits within the first six months of treatment

Using log-binomial regression, we identified no strong independent predictors of missing a medical or ARV visit in the first six months on ART. We found that males, younger patients and those with BMI of <17.5 had a somewhat increased risk of missing a visit in the first six months (Table 2). Patients on an AZT-based regimen (vs. d4T) (RR 1.1; 95% CI 0.8-1.4) and those on a NVP-based regimen (vs. EFV) (RR 1.2; 95% CI 1.0-1.4) had an increased risk of missing a medical visit early on in treatment. While not perfectly consistent, baseline CD4 count was not predictive of missing a medical or ARV visit during the first six months of ART.

**Table 2:** Crude and adjusted predictors of missing a medical or ARV visit in the first six months on ART in Johannesburg, South Africa (n = 4476)

		Medical visits		ARV visits	
**Variable**		**Crude RR** **(95% CI)**	**Adjusted RR^†^** **(95% CI)**	**Crude RR** **(95% CI)**	**Adjusted RR^†^** **(95% CI)**

Sex	Female	Reference	Reference	Reference	Reference
	Male	1.15 (1.05-1.27)	1.16 (1.05-1.28)	1.14 (1.03-1.28)	1.15 (1.02-1.28)
					
Baseline BMI	≥17.5	Reference	Reference	Reference	Reference
	< 17.5	1.11 (1.00-1.24)	1.09 (0.97-1.22)	1.24 (1.09-1.40)	1.22 (1.08-1.39)
					
Age at ART initiation	≥ 50	Reference	Reference	Reference	Reference
	40-49.9	1.11 (0.92-1.35)	1.15 (0.94-1.40)	1.00 (0.80-1.25)	1.00 (0.80-1.26)
	30-39.9	1.04 (0.87-1.25)	1.07 (0.89-1.30)	1.08 (0.88-1.33)	1.08 (0.87-1.33)
	25-29.9	1.07 (0.87-1.32)	1.10 (0.89-1.37)	1.01 (0.80-1.29)	1.03 (0.81-1.32)
	18-24.9	1.10 (0.83-1.45)	1.13 (0.86-1.51)	1.22 (0.91-1.65)	1.23 (0.90-1.67)
					
Baseline CD4 count (cells/mm^3^)	100-200	Reference	Reference	Reference	Reference
	51-100	0.89 (0.78-1.01)	0.88 (0.77-1.00)	0.93 (0.81-1.08)	0.95 (0.83-1.07)
	0-50 vs.	1.00 (0.90-1.12)	0.98 (0.88-1.09)	0.99 (0.88-1.12)	0.93 (0.80-1.07)
					
Baseline Hb (ug/dL)	≥ 10.0	Reference	Reference	Reference	Reference
	< 10.0	1.01 (0.91-1.13)	1.02 (0.92-1.14)	1.00 (0.89-1.14)	1.00 (0.88-1.13)
					
Baseline WHO stage	I/II	Reference	Reference	Reference	Reference
	III/IV	1.04 (0.94-1.14)	1.03 (0.93-1.14)	1.06 (0.95-1.19)	1.03 (0.92-1.16)
					
Baseline NRTI	d4T	Reference	Reference	Reference	Reference
	AZT	1.13 (0.86-1.48)	1.10 (0.84-1.44)	0.99 (0.71-1.39)	0.98 (0.70-1.38)
					
Baseline NNRTI	EFV	Reference	Reference	Reference	Reference
	NVP	1.13 (0.96-1.33)	1.18 (1.00-1.40)	1.04 (0.85-1.26)	1.05 (0.86-1.29)

†Relative risks (RR) are from a log-binomial regression model also adjusted for baseline BMI, age, baseline CD4, baseline haemoglobin, baseline WHO stage and baseline regimen

CI, confidence interval; ART, antiretroviral therapy; BMI, body mass index; Hb, haemoglobin; d4T, stavudine; AZT, zidovudine; EFV, efavirenz; NVP, nevirapine; NRTI, nucleoside reverse transcriptase inhibitor; NNRTI, non-nucleoside reverse transcriptase inhibitor

### CD4 count response and viral load suppression

In adjusted models, we found that patients who missed three or more medical visits had the highest risk of poor CD4 response by six months on ART (RR 2.3; 95% CI: 1.4-3.8) (Table 3). Male patients and those on AZT-based regimens (vs. d4T) were also at increased risk of poor CD4 response, while patients younger than 50 years of age were less likely to have had a poor CD4 response in the first six months on ART compared with patients 50 years of age or older. Patients who missed 2 and ≥3 medical (RR 2.3; 95% CI: 1.5-3.4 and RR 3.0; 95% CI: 1.3-7.1, respectively) or ARV visits (RR 1.9; 95% CI: 1.1-3.3 and RR 5.8; 95% CI: 3.0-11.1, respectively) were at increased risk of failing to achieve viral suppression by six months on treatment compared with patients who attended all scheduled visits. Although not significant, patients younger than 25 years of age and those on an AZT-based regimen (vs. d4T) or NVP-based regimen (vs. EFV) were also at increased risk of failing to achieve viral load suppression by six months.

**Table 3:** Relationship between missing an HIV treatment visit in the first six months of ART and poor CD4 response and failure to suppress viral load in Johannesburg, South Africa (n = 4476)

Visits	ARV		Medical visits	
**Poor CD4 response**^¥^				

	**Crude RR** **(95% CI)**	**Adjusted RR^£^** **(95% CI)**	**Crude RR** **(95% CI)**	**Adjusted RR^£^** **(95% CI)**

Missed visit status				
1 vs. 0	1.02 (0.88-1.19)	1.02 (0.87-1.18)	1.15 (1.00-1.33)	1.12 (0.98-1.29)
2 vs. 0	1.36 (0.99-1.85)	1.33 (0.98-1.80)	1.22 (0.90-1.65)	1.19 (0.89-1.61)
≥ 3 vs. 0	1.24 (0.37-4.14)	1.62 (0.49-5.34)	2.11 (1.91-3.74)	2.27 (1.35-3.83)
				
Age at initiation				
18-24.9 vs. ≥50	0.46 (0.31-0.69)	0.52 (0.35-0.78)	0.46 (0.31-0.69)	0.52 (0.34-0.77)
25-29.9 vs. ≥50	0.56 (0.43-0.72)	0.62 (0.48-0.79)	0.56 (0.43-0.72)	0.61 (0.48-0.79)
30-39.9 vs. ≥50	0.69 (0.57-0.83)	0.73 (0.61-0.88)	0.69 (0.57-0.83)	0.73 (0.60-0.88)
40-49.9 vs. ≥50	0.82 (0.67-1.01)	0.84 (0.69-1.02)	0.82 (0.67-1.01)	0.83 (0.68-1.02)
				
Baseline CD4 count (cells/mm^3^)				
51-100 vs. 100-200	0.76 (0.65-0.89)	0.76 (0.65-0.89)	0.76 (0.65-0.89)	0.77 (0.64-0.88)
0-50 vs. 100-200	0.68 (0.59-0.78)	0.68 (0.59-0.79)	0.68 (0.59-0.78)	0.68 (0.60-0.79)
				
Baseline NRTI				
AZT vs. d4T	1.41 (0.80-2.49)	1.42 (0.81-2.50)	1.41 (0.80-2.49)	1.46 (0.83-2.57)
				
Baseline NNRTI				
NVP vs. EFV	0.84 (0.66-1.08)	0.92 (0.72-1.18)	0.84 (0.66-1.08)	0.93 (0.73-1.19)
				
Sex				
Male vs. female	1.40 (1.24-1.58)	1.33 (1.18-1.51)	1.40 (1.24-1.58)	1.32 (1.17-1.50)

**Failure to suppress viral load**^€^				

Missed visit status				
1 vs. 0	1.15 (0.89-1.50)	1.19 (0.89-1.51)	1.24 (0.97-1.58)	1.23 (0.96-1.57)
2 vs. 0	2.09 (1.33-3.29)	1.93 (1.12-3.32)	2.18 (1.47-3.24)	2.28 (1.54-3.36)
≥ 3 vs. 0	4.35 (1.71-11.0)	5.75 (2.97-11.1)	3.19 (1.34-7.58)	3.00 (1.28-7.06)
				
Age at initiation				
18-24.9 vs. ≥50	1.64 (0.98-2.74)	1.55 (0.90-2.73)	1.64 (0.98-2.74)	1.41 (0.83-2.38)
25-29.9 vs. ≥50	1.13 (0.73-1.77)	1.01 (0.62-1.64)	1.13 (0.73-1.77)	0.96 (0.61-1.52)
30-39.9 vs. ≥50	0.98 (0.66-1.46)	0.99 (0.65-1.50)	0.98 (0.66-1.46)	0.92 (0.62-1.36)
40-49.9 vs. ≥50	0.92 (0.60-1.40)	0.93 (0.58-1.47)	0.92 (0.60-1.40)	0.85 (0.56-1.31)
				
Baseline CD4 count (cells/mm^3^)				
51-100 vs. 100-200	0.97 (0.74-1.20)	0.96 (0.70-1.30)	0.97 (0.74-1.20)	0.99 (0.75-1.30)
0-50 vs. 100-200	0.93 (0.74-1.29)	0.94 (0.72-1.24)	0.93 (0.74-1.29)	0.92 (0.72-1.19)
				
Baseline NRTI				
AZT vs. d4T	1.41 (0.80-2.49)	1.69 (0.93-3.05)	1.41 (0.80-2.49)	1.60 (0.93-2.75)
				
				
Baseline NNRTI				
NVP vs. EFV	0.79 (0.56-1.12)	1.12 (0.75-1.67)	0.79 (0.56-1.12)	1.22 (0.86-1.73)
				
Sex				
Male vs. female	0.81 (0.65-1.01)	0.80 (0.60-1.04)	0.81 (0.65-1.01)	0.85 (0.67-1.07)

£ Relative risks (RR) are from a log-binomial regression model also adjusted for baseline haemoglobin, baseline WHO stage and baseline body mass index

¥ Poor CD4 response defined as an increase of <50 cells/mm3 by six months on ART

€ Failure to suppress viral load defined as a viral load ≥400 copies/ml by six months on ART

CI, confidence interval; ART, antiretroviral therapy; d4T, stavudine; AZT, zidovudine; EFV, efavirenz; NVP, nevirapine; NRTI, nucleoside reverse transcriptase inhibitor; NNRTI, non-nucleoside reverse transcriptase inhibitor

### Mortality and loss to follow up

Adjusted multivariable Cox proportional hazards analysis showed that more missed visits were generally associated with increased risk of death and loss to follow up over 12 months of follow up (which began nine months after ART initiation) (Table 4). In terms of mortality, patients who missed 2 and ≥3 medical visits had more than a two- to four-fold increased risk. The risk increased to eight-fold (HR 8.2; 95% CI: 2.0-33.7) for patients who missed ≥3 ARV visits, although it was estimated with wide confidence intervals. We saw no increased risk of death among those who missed one medical or ARV visit versus those who missed none.

**Table 4:** Crude and adjusted hazard ratios of death and loss to follow up stratified by missed visit status among ART patients in Johannesburg, South Africa (n = 4476)

Visit type	No. Events (%)	Person time (years)	Rate (per 100 person years)	Crude HR (95% CI)	Adjusted HR‡ (95% CI)
**Death**^§^					

Medical visits					
0	76 (2.3%)	3080.6	2.5	Reference	Reference
1	28 (2.7%)	984.4	2.8	1.15 (0.75-1.78)	1.11 (0.72-1.72)
2	8 (4.9%)	152.2	5.3	2.12 (1.03-4.40)	2.06 (1.00-4.28)
≥ 3	3 (15.0%)	15.7	19.1	7.69 (2.43-24.4)	4.74 (1.39-16.2)
					
ARV visits					
0	90 (2.6%)	3309.3	2.7	Reference	Reference
1	23 (2.7%)	806.5	2.9	1.05 (0.66-1.66)	1.02 (0.65-1.62)
2	0 (0%)	107.8	0	-	-
≥ 3	2 (18.2%)	9.3	21.5	7.87 (1.94-32.0)	8.15 (1.97-33.7)

**Lost to follow up**^^^					

Medical visits					
0	196 (6.0%)	3080.6	6.4	Reference	Reference
1	62 (5.9%)	984.4	6.3	0.99 (0.75-1.32)	0.97 (0.73-1.30)
2	15 (9.1%)	152.2	9.9	1.55 (0.92-2.63)	1.54 (0.91-2.61)
≥ 3	4 (22.0%)	15.7	25.5	4.07 (1.51-10.9)	2.98 (1.10-8.14)
					
ARV visits					
0	196 (5.6%)	3309.3	5.9	Reference	Reference
1	57 (6.6%)	806.5	7.1	1.20 (0.89-1.61)	1.20 (0.89-1.61)
2	24 (19.8%)	107.8	22.3	3.80 (2.48-5.80)	3.81 (2.48-5.84)
≥ 3	0 (0%)	9.3	0	-	-

‡ Hazard ratios (HR) are from a Cox proportional regression models adjusted for age, sex, total number of scheduled visits, baseline regimen and CD4 count after nine months on ART

§ Death obtained from South African National Vital Registration Infrastructure Initiative

^Lost to follow up defined as ≥4 months since last visit

CI, confidence interval

Patients who missed 2 and ≥3 medical or ARV visits also had a nearly two- to four-fold increased risk of becoming lost during the follow up period. As with death, missing one medical or ARV visit was not strongly associated with an increased risk of becoming lost to follow up.

## Discussion

Recent estimates state that 5.2 million HIV-positive people have initiated ART in resource-limited settings, an increase of 1.2 million since the end of 2008 [27]. Initiating this many patients onto care in such a short time is a remarkable achievement, but is not without its challenges. Keeping these patients in care and adherent to treatment, especially in the early stages of ART, is challenging the world over, and appears to be particularly difficult in resource-limited settings. In our study, more than 35% of patients attending the Themba Lethu Clinic in Johannesburg, South Africa, failed to attend at least one clinic visit on time in the first six months of treatment. This finding is consistent with previous reports, from industrialized countries, documenting 25%-44% of recently diagnosed HIV-infected individuals failing to adhere to scheduled visits early on in their care or treatment [28-32]. Close to 10% of the cohort missed two or more medical or ARV visits in the first six months of ART, and this was strongly associated with negative outcomes.

Documenting the amount of missed visits early on in treatment is critical because of its potential implications for poorer treatment outcomes. Previous studies have shown that patients who miss visits soon after initiating ART are at increased risk of early mortality and loss [16,17]. In our study, patients who were unable to adhere to clinic visits early on in treatment, but returned to care, were at increased risk of poorer ART outcomes (death and loss to follow up) over 12 months of follow up beginning after a full nine months on treatment, particularly if they missed more than one visit. This suggests that missing visits early is a marker for those who will have poorer outcomes even if returning to care. Our data are not able to elucidate the specific mechanisms by which missing visits lead to poorer outcomes, but it is likely that this is a marker for poor adherence.

Our results show that patients who miss visits early on in care are less likely to achieve virologic suppression after six months of ART compared with patients who attended all scheduled visits. As adherence is critical to the success of their treatment [11-13], missing medical or ARV collection visits could compromise the continuous supply of ART required to achieve long-term viral suppression. Poor adherence to treatment as a result of missing visits could result in failure to initially suppress the virus [14,15,17,20] or cause a viral load rebound [19], putting an individual at risk of drug resistance [13]. Our results also support previous research showing that older patients mount poorer CD4 cell count responses [33], but are more likely to adhere to clinic visits [34] and achieve virologic suppression [33,34] compared with their younger counterparts.

Alternatively, some of the relationship between missed visits and poor treatment outcomes may result from missed opportunities to detect and treat opportunistic infections. These conditions may still be diagnosed and treated at a later time, but opportunistic infections after initiation of ART are associated with poor immune recovery [35,36]. A longer period of time spent at a lower CD4 count has been shown to increase patients' overall risk of mortality [36,37]. Our finding that patients who miss visits early on in care have a poorer CD4 response supports this point further.

The reasons that patients miss visits early on in care are likely to be multi-factorial and complex. While we found no strong predictors of missing medical visits, our findings show that male gender, younger age, low baseline BMI, AZT-based versus d4T- and NVP-based versus EFV-based regimens are predictive of missing visits early on in treatment. Data from industrialized countries has shown that younger patients [14,28,29,38], those with poorer education [38] and those with low socio-economic status [29] were more likely to miss visits related to their HIV, but there is little evidence from resource-limited settings. While not perfectly consistent [18], most studies on the topic have shown that healthier patients were more likely than sicker ones to miss scheduled appointments [28-31]. Surprisingly, we did not detect a relationship between CD4 count and missed visits in our population. Distrust in the healthcare system, stigmatization of those infected by their communities and patient financial constraints could also play a major role in how adherent patients are to visit schedules in a resource-limited setting [39]. In sub-Saharan Africa, the cost of seeking care, even when treatment is provided at no cost, can be an important barrier to staying in care [40].

Our study is one of the first to report on the specific relationship between missed visits in the first six months of ART and clinical HIV treatment outcomes in a resource-limited setting and longer term outcomes. Still, our findings should be considered alongside the study's limitations. First, because our study reports data from a single government HIV clinic, our results may not be generalizable to the overall population. Second, data on specific reasons for missed visits is not collected and therefore we could not determine what the major barriers are to maintaining a consistent schedule of care and treatment.

Finally, we did not have actual ARV drug dispensing data, and while missing scheduled drug collection visits is likely to mean that a patient is without medication, we cannot rule out the possibility that the individuals were dispensed additional months of drug supplies. However, in this study we considered only visits in the first six months after treatment initiation, a period when the pharmacy is least likely to dispense several months of medication. This is also a period when a patient needs to see a clinician in order to have his or her ARV prescription renewed, and so we anticipate that this occurred infrequently.

## Conclusions

Our results suggest that patients who miss multiple visits in the earliest period of treatment but remain in care are at increased risk of poorer ART outcomes. Currently, a substantial proportion of funding and manpower in resource-limited settings is focused on reducing mortality and loss to follow up among those started on ART. Our findings suggest that targeting those who miss visits early on in treatment could have benefits in terms of longer term mortality reductions. Future studies need to focus on identifying the barriers to adhering to a visit schedule so that interventions and support services can be directed at those at risk.

## Competing interests

MM and IS are affiliated to Right to Care, which provided some of the funding for the current research and also supports the provision of treatment for the patients in the study. All authors (Alana Brennan, Mhairi Maskew, Ian Sanne and Matthew P Fox) have no other competing interests to declare.

## Authors' contributions

AB contributed to the study concept and design and to analysis and interpretation of the data, in addition to drafting and critical revision of the manuscript for important intellectual content. MM contributed to the analysis and interpretation of the data and critical revision of the manuscript. IS contributed to the acquisition of data, provided critical revision of the manuscript and obtained funding. MF contributed to the study concept and design and to analysis and interpretation of the data, in addition to drafting and critical revision of the manuscript for important intellectual content. All authors have read and approved the final manuscript.
